# Mechanisms of transcriptional regulation in *Anopheles gambiae* revealed by allele-specific expression

**DOI:** 10.1098/rspb.2024.1142

**Published:** 2024-09-18

**Authors:** Naomi A. Dyer, Eric R. Lucas, Sanjay C. Nagi, Daniel P. McDermott, Jon H. Brenas, Alistair Miles, Chris S. Clarkson, Henry D. Mawejje, Craig S. Wilding, Marc S. Halfon, Hasiba Asma, Eva Heinz, Martin J. Donnelly

**Affiliations:** ^1^ Department of Vector Biology, Liverpool School of Tropical Medicine, Pembroke Place, Liverpool L3 5QA, UK; ^2^ Wellcome Sanger Institute, Wellcome Genome Campus, Hinxton, Cambridge CB10 1SA, UK; ^3^ Infectious Diseases Research Collaboration (IDRC), Plot 2C Nakasero Hill Road, PO Box 7475, Kampala, Uganda; ^4^ School of Biological and Environmental Sciences, Liverpool John Moores University, Byrom Street, Liverpool L3 3AF, UK; ^5^ Department of Biochemistry, Jacobs School of Medicine & Biomedical Sciences, University at Buffalo-State University of New York, 955 Main Street, Buffalo, NY 14203, USA; ^6^ Strathclyde Institute of Pharmacy & Biomedical Sciences, University of Strathclyde, Glasgow G4 0RE, UK; ^7^ Department of Clinical Sciences, Liverpool School of Tropical Medicine, Pembroke Place, Liverpool L3 5QA, UK

**Keywords:** allele, *cis*-regulation, transcript, insecticide, resistance

## Abstract

Malaria control relies on insecticides targeting the mosquito vector, but this is increasingly compromised by insecticide resistance, which can be achieved by elevated expression of detoxifying enzymes that metabolize the insecticide. In diploid organisms, gene expression is regulated both in *cis*, by regulatory sequences on the same chromosome, and by *trans* acting factors, affecting both alleles equally. Differing levels of transcription can be caused by mutations in *cis*-regulatory modules (CRM), but few of these have been identified in mosquitoes. We crossed bendiocarb-resistant and susceptible *Anopheles gambiae* strains to identify *cis*-regulated genes that might be responsible for the resistant phenotype using RNAseq, and CRM sequences controlling gene expression in insecticide resistance relevant tissues were predicted using machine learning. We found 115 genes showing allele-specific expression (ASE) in hybrids of insecticide susceptible and resistant strains, suggesting *cis*-regulation is an important mechanism of gene expression regulation in *A. gambiae*. The genes showing ASE included a higher proportion of *Anopheles*-specific genes on average younger than genes with balanced allelic expression.

## 1. Introduction

Malaria prevalence in Sub-Saharan Africa has reduced by 50% since 2000, primarily due to insecticide-based control of mosquito vectors [1]. Recently, progress has stagnated [2], partly due to increasing levels of resistance against insecticides in mosquito populations [3]. *Anopheles gambiae* is one of the dominant malaria vectors in Sub-Saharan Africa, the primary vector across most of Uganda [4] and the vector for which the largest resource of genome data is available, with 7275 genomes sequenced [5–8]. A common cause of insecticide resistance is increased degradation of insecticides (termed metabolic resistance) [9] with overexpression of insecticide-metabolizing P450s repeatedly implicated [10–12]. This can be caused by mutations in *cis*-regulatory regions regulating the expression of metabolic resistance genes. In diploid organisms, such mutations typically only affect the expression of the allele of the gene located on the same chromosome. Although some *trans* factors involved in metabolic resistance gene regulation in *Anopheles* are known [13,14], few studies have identified genetic variation causing metabolic resistance [15–18]. The multiallelic nature of metabolic insecticide resistance which can involve different mutations affecting the same gene in different populations, as well as the involvement of multiple genes, makes marker identification challenging as it limits the power of association studies unless very large sample sizes are used [15].

Despite the primary role of gene overexpression in metabolic resistance, only one *cis*-regulatory variant for resistance-linked differential expression has been identified in *A. gambiae* [19], and markers for such variants are therefore absent in the current genetic marker panel for resistance [20]. Copy number variants (CNV) have been observed in *A. gambiae* metabolic resistance gene clusters [21]. For example, in *Anopheles coluzzii* copy number of *Cyp6AA1* is associated with deltamethrin resistance [22]. The relative contribution of CNV and *cis*-regulation on *Anopheles* gene expression has not yet been determined.

Uganda sees a high burden of malaria; comprising 7.8% of all global cases in 2021 [23]. To address this public health burden, the insecticide bendiocarb has been used for indoor residual spraying (IRS) to complement the distribution of long-lasting insecticidal nets. Some resistance to bendiocarb in *A. gambiae* was observed in Nagongera (southeast Uganda) and Kihihi (southwest Uganda), with 83% and 70% mortality, respectively, to World Health Organization (WHO) bioassays with a diagnostic dose of 0.1% bendiocarb in 2014 prior to the IRS [24]. The IRS campaign starting in December 2014 succeeded in reducing the human biting rate, cases of malaria and test positivity rate in Nagongera [24], but the potential for further increases in resistance puts the long-term usefulness of bendiocarb into question.

Mosquitoes collected from Nagongera in 2014, which have moderate resistance to bendiocarb, showed significant differential expression of many genes compared with the susceptible Kisumu strain, including salivary gland protein encoding *D7r2* and *D7r4* genes as well as the detoxification-associated genes *Gstd3* and *Cyp6m2* [25]. Expression of *D7r4* was associated with a single-nucleotide polymorphism (SNP) in a non-coding transcript downstream of the D7 cassette [25].

In diploid organisms, allele-specific expression (ASE) provides strong evidence that genes may be under differential *cis*-regulatory control [26,27]. Using a method that has been applied to a variety of taxa [26,28–32] but not mosquitoes, we describe the identification of genes showing ASE in *A. gambiae* which potentially confer metabolic resistance. The challenges of applying this method that arise from mosquito biology and genome structure are discussed. In complementary work, we predicted the sequences of some of the *cis*-regulatory modules (CRM) that may underlie the expression of genes involved in insecticide resistance using machine learning. Predictions included potential CRMs proximal to the genes showing ASE and genes that show consistent differential expression patterns in multiple resistant *Anopheles* strains, providing a starting point for future investigations into CRM variants during the evolution of insecticide resistance.

## 2. Material and methods

### (a) Strains and crosses

Resting female mosquitoes were collected in Siwa Village, Nagongera, Tororo District in Eastern Uganda (0°46′12.0″N, 34°01′34.0″E) in March 2013. Sixty-five *A. gambiae* (as ascertained by species ID PCR [33]) egg batches laid by the collected females were reared and combined to establish the Nagongera colony. All colony founding mothers were screened for the bendiocarb target site resistance mutation G280S in *ace1* using the TaqMan assay described by Bass *et al.* [34]. No resistance-associated variants were observed. Full sequencing of *ace1* from six colony mosquitoes that survived bendiocarb exposure did not reveal any other potential target site resistance mutations. The colony was assayed using WHO tube assays [35] at F2 and found to be highly resistant to DDT (7.4% mortality to diagnostic dose 4%, *n* = 54) and deltamethrin (17.2% mortality to diagnostic dose 0.05%, *n* = 64), with intermediate resistance to the carbamate bendiocarb (65.9% mortality to diagnostic dose 0.1%, *n* = 44). WHO tube bioassays [35,36] of Nagongera colony adults using assays with preexposure to 4% piperonyl butoxide (which inhibits P450-mediated metabolism) for 1 h prior to bendiocarb exposure increased mortality to 97.4% (*n* = 39), a 48% increase in mortality compared with bendiocarb exposure alone, implying that bendiocarb resistance is metabolic rather than target site mediated.

**Figure 1.**
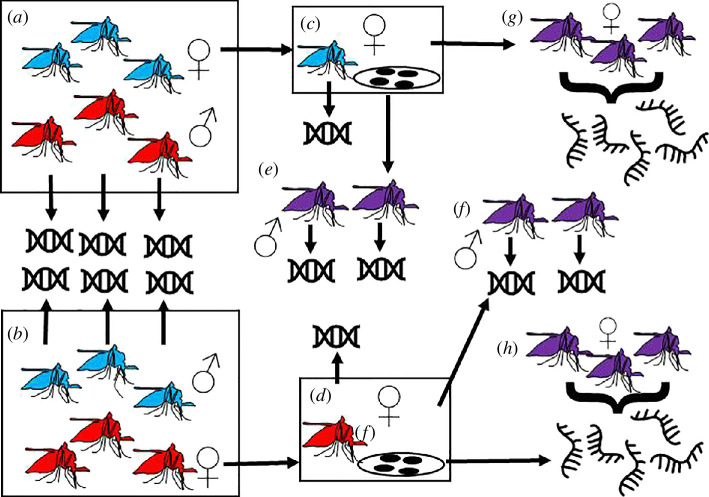
Crossing, DNA and RNA extraction schema. (*a*) Thirteen Kisumu females (blue) were crossed to 13 Nagongera males (red). Females mate only once. Genomic DNA from Nagongera males was extracted and sequenced. (*b*) In the reciprocal cross, 13 Nagongera females (red) were crossed to 13 Kisumu males (blue). Genomic DNA was extracted and sequenced from Kisumu males. (*c*) Individual mated Kisumu females were transferred to laying cups. Genomic DNA was extracted and sequenced following egg laying. (*d*) Individual mated Nagongera females were transferred to laying cups. Genomic DNA was extracted and sequenced following egg laying. (*e*) F1 progeny (purple) from the three Kisumu mothers sequenced at step (*c*) were raised to adulthood. Genomic DNA was extracted and sequenced from individual males (*f*). F1 progeny (purple) from the three Nagongera mothers sequenced at step (*d*) were raised to adulthood. Genomic DNA was extracted and sequenced from individual males (*g*,*h*). Female F1 from each of the six mothers were raised to adulthood. RNA was extracted and sequenced from pools of 10 F1 females 3–5 days after eclosion.

A schematic of the experimental design is shown in figure 1. Reciprocal crosses between Nagongera mosquitoes and an inbred insecticide susceptible colony (origin Kisumu, Kenya, 1975, susceptible to bendiocarb, DDT and deltamethrin) were performed as described previously [6]. Crosses each involved 13 males and multiple females; since *A. gambiae* are swarm maters, multiple males are required to induce mating. Females were transferred to individual cups for egg laying. F1 progeny of three females from each reciprocal cross were raised to adults under standard insectary conditions of 12 h light : 12 h dark cycle, 26°C ± 2°C and 70% relative humidity and fed on 10% sucrose solution (table 1, figure 1).

**Table 1.** Crosses between Kisumu and Nagongera and RNAseq summary statistics.

RNAseq sample	cross	mother origin	mother Sanger code	father origin	father Sanger code	total reads counted (F1 RNAseq)	percentage of reads mapping to *A. gambiae* PEST genome
Wilding_1	B1	Nagongera	failed QC	Kisumu	unknown	18 496 263	88.6%
Wilding_2	B3	Nagongera	failed QC	Kisumu	unknown	38 526 717	87.1%
Wilding_3	B5	Nagongera	AC0382-C	Kisumu	AC0416-C	28 969 694	87.7%
Wilding_4	K2	Kisumu	AC0300-C	Nagongera	AC0406-C	6 400 460	85.5%
Wilding_5	K4	Kisumu	AC0317-C	Nagongera	AC0398-C	46 229 505	86.3%
Wilding_6	K6	Kisumu	AC0334-C	Nagongera	AC0398-C	29 491 773	86.9%

### (b) Sequencing

RNA was extracted from pools of 10 female F1 progeny from each of the six crosses 3–5 days after eclosion using RNAqueous4PCR total RNA isolation kit (Invitrogen). RNA quality and quantity were checked using the Agilent Bioanalyser profile and Qubit 2.0 Fluorometer. Total RNA libraries were prepared for Illumina paired-end indexed sequencing according to the Illumina TruSeq RNA sample preparation v. 2 guide [37]. cDNA libraries were barcoded, pooled and sequenced using the Illumina HiSeq1500 platform, with 100 bp paired-end reads.

DNA was extracted using the Qiagen DNeasy Kit from the six individual mothers, all individual potential fathers alive following the cross and individual male F1 siblings (figure 1) and sequenced as described previously [6].

### (c) Genome analysis

Quality control of genomic sequences for mothers, potential fathers and F1 progeny and matching of fathers to the progeny using Mendelian error analysis was performed as described previously [6], electronic supplementary material, table S1.

Scikit-allel v. 1.3.7 [38] was used to analyse the genome of the mothers and potential fathers, generating a consensus sequence of homozygous sites for Kisumu and Nagongera colonies and calculating the number of SNPs differing between the colonies which passed the MalariaGen ‘gamb_colu’ site filter (table 2). Analysis of CNV, selective sweeps, protein age and enrichment analysis are described in electronic supplementary materials.

**Table 2.** Number of SNPs differing between Kisumu and Nagongera colony consensus genome sequences and SNPs in exonic regions differing between the parents that could be used to detect ASE

Cross or Colony	2L	2R	3L	3R	X	Y / other	Total
Colony consensus	2519	12 769	1530	1931	10 510	0	29 259
K2	13 849	30 134	19 375	20 877	15 543	0	99 778
K4	33 139	35 048	19 074	22 405	15 250	0	124 916
K6	30 303	30 811	14 534	17 801	15 601	2	109 052
B5	29 627	36 203	20 865	25 414	10 539	1	122 649

Colony consensus numbers are the count of SNPs on each chromosome arm which differ between the Kisumu and Nagongera colony and are homozygous in all sequenced Kisumu and Nagongera colony individuals. Numbers for each cross are exonic SNPs that are homozygous in the parents of each cross but differing between the parents

### (d) RNAseq analysis

Samples were compared using the RNA-Seq-Pop snakemake workflow [39] which includes FastQC [40], read alignment to the *A. gambiae* PEST reference genome (AgamP4, INSDC Assembly GCA_000005575.1, February 2006) using kallisto [41], principal components analysis, differential gene expression analysis using DESeq2 [42], differential isoform analysis using sleuth [43] and gene set enrichment analysis using GSEA [44]. RNAseq data for the Kisumu parental colony, Busia G28 deltamethrin selected colony [39] and Tiefora pyrethroid resistant colony [45] were compared with the F1 RNAseq data.

### (e) Analysis of ASE

For parent-based mapping, SNPs homozygous for different alleles in the parents were used to infer ASE. Due to the pooling of 10 females for RNA sequencing, it was not possible to use parental heterozygous SNPs. For sibling-based mapping, autosomal SNPs distinguishing maternal and paternal alleles were inferred from the male siblings’ genome sequences (electronic supplementary material, tables S2 and S3).

Analysis of F1 RNAseq data for ASE was performed using ASEReadCounter* [46] (based on ASEReadCounter [47]) using individual parent genomes for each cross to assign reads to parental alleles (options –vcf_mat, –vcf_pat). Where only sibling-inferred SNPs were known, option –vcf_joind was used on the unphased inferred F1 VCF file. SNP level ASE was calculated for all SNPs with ≥10 total counts.

To obtain a gene level measurement of ASE, MBASED [48] was used to aggregate SNP level counts to gene level by pseudo-phasing SNPs in each gene. The SNPs with the higher read count at each variable site are combined into the major allele. For SNPs inferred from siblings, MBASED was run assuming SNPs were unphased. Where phase was known, MBASED was run in both phased and non-phased mode to measure the impact on power to detect loci with ASE. The overdispersion parameter for simulations was determined from read counts (electronic supplementary materials). In all cases, the same seed was set prior to running 10^6^ simulations with the total read count at each SNP kept constant, but drawing reference allele counts from a null beta-binomial distribution with mean 0.5 × total count and overdispersion parameter 0.038. The *p*-value is the proportion of simulations in which the major allele frequency (MAF) is greater than or equal to the observed MAF. For genes with multiple SNPs, the *p*-value for heterogeneity (*p*_het_) between the level of ASE at each SNP was calculated. Low *p*_het_ indicates possible isoform-specific ASE. False discovery rate correction was applied to both *p*-values for ASE and *p*_het_ with a nominal rate of 5% [49].

### (f) Prediction of CRMs

*Anopheles* CRMs were predicted using SCRMshaw [50–54]. Briefly, *Drosophila* CRMs which drive gene expression in the tissue of interest were downloaded from the Redfly database (v. 9.6.0, database updated 2 January 2023) [55,56], with max size 2000. Only non-overlapping sequences (>100 bp) were included. Syntenous regions from related *Drosophila* species (putatively containing the equivalent CRM) were added to the training dataset using liftOver at https://genome.ucsc.edu/cgi-bin/hgLiftOver [57] as described by Kazemian & Halfon [51]. SCRMshaw was trained using this augmented training set and a 10× bigger set of non-CRM non-exonic regions. Repeats in CRMs, non-CRMs and the target *A. gambiae* PEST genome were masked using repeat masker [58]. Existing training sets for the adult peripheral nervous system, embryonic and larval excretory and embryonic/larval Malpighian tubules were downloaded from GitHub (https://github.com/HalfonLab/dmel_training_sets). These were compared with the 2272 Tn5 transposase hypersensitive sites identified by Ruiz *et al.* [59].

## 3. Results and discussion

### (a) Crosses

Three females from each reciprocal cross between the Kisumu and Nagongera strains laid viable eggs (figure 1 and table 1). For four crosses the father was identified by minimizing median Mendelian error (electronic supplementary material, table S1), but for the other two crosses the mother failed sequencing quality control and none of the sequenced putative fathers were a good match. We presume the true fathers for these crosses died during the experiment, precluding extraction of good quality DNA.

RNAseq statistics for the pooled F1 females from each of these six crosses are shown in table 1. The number of reads recommended for 60% power to detect ASE at 1.5-fold is 500 per gene [60], which for the 13 796 annotated genes in *A. gambiae* would require around 6.9 × 10^6^ reads. Five crosses exceeded this value with one cross, K2, having slightly fewer (6.4 × 10^6^ reads). The proportion of reads mapping to the *A. gambiae* genome was similar for all crosses (mean 87.0%, s.d. 1.1%).

### (b) Between pools gene expression comparison

**Figure 2.**
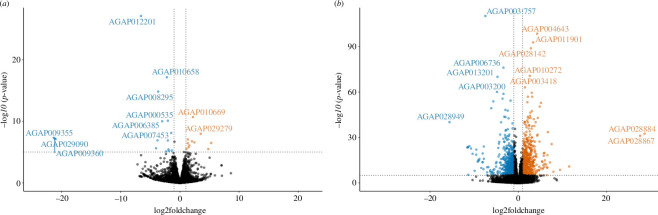
Reciprocal crosses show similar overall gene expression. (*a*) Volcano plot of log_2_ fold change against −log_10_ *p*-value comparing gene expression in the F1 progeny of reciprocal crosses between Kisumu and Nagongera strains. Blue points: genes downregulated in progeny of Kisumu mothers; orange points: genes upregulated in progeny of Kisumu mothers. (*b*) Volcano plot of log_2_ fold change against −log_10_ *p*-value comparing gene expression between F1 progeny of Nagongera and Kisumu with the Kisumu parental strain. Blue points: genes downregulated in the Kisumu compared with cross progeny; orange points: genes upregulated in Kisumu compared with cross progeny.

Comparison between reciprocal crosses revealed similar gene expression in the F1 progeny, with just nine genes significantly downregulated and 12 genes significantly upregulated in the F1 progeny of Kisumu mothers compared with F1 progeny of Nagongera mothers at *p*_adj_ ≤ 0.001 (figure 2*a*). When comparing only F1 crosses in PCA, B1 seemed to be an outlier in terms of expression (electronic supplementary material, figure S1a). However, when compared with other colonies of *A. gambiae* s.l., the F1 progeny formed a distinct group (electronic supplementary material, figure S1b).

F1 gene expression differed from the Kisumu (parental) colony, with a total of 1362 genes upregulated and 1265 genes downregulated in the F1 compared to the Kisumu colony (*p*-adj ≤ 0.001) (figure 2*b*). Differential isoform expression results are available at Dryad [61]. RNAseq data were unavailable for the Nagongera colony, which no longer exists. Gene set enrichment analysis of the differentially expressed genes indicated that genes with Gene Ontology (GO) terms associated with odorant binding, olfactory receptor activity, sensory perception of smell, response to stimulus, detection of chemical stimulus involved in sensory perception of smell, structural constituent of cuticle and signal transduction were downregulated in the F1 compared with Kisumu, whereas those with GO terms associated with translation, ribosome, mitochondrion, structural component of ribosome and serine type endopeptidase activity were upregulated in the F1 compared with Kisumu (all at *p*-adjusted 0.05). KEGG pathway analysis showed significant upregulation of ribosome, citrate cycle (TCA cycle) and oxidative phosphorylation in the F1 compared with Kisumu, and no KEGG pathway significantly downregulated.

### (c) ASE inference

**Figure 3.**
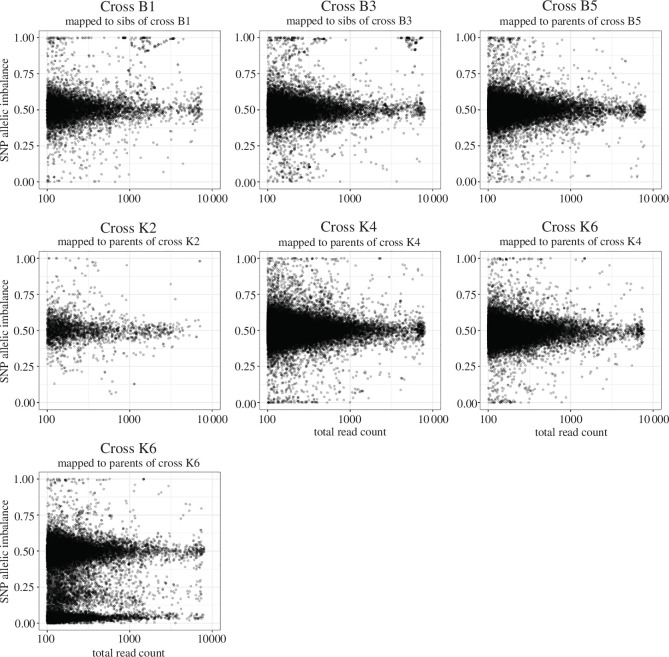
ASE in progeny of crosses between strains. Plots of total read count against ASE at each SNP for SNPs with at least 100 reads. Cross and source of SNPs used to count reads are indicated at the top of each plot. For crosses B5, K2 and K6 the SNP ASE indicates maternal reads/total reads at SNP, and for crosses B1 and B3 SNP ASE indicates reference reads/total reads at SNP. Cross K6 is shown with ASE inferred using SNPs from both the parents of cross K4 and for the initially assumed parents of cross K6.

Detection of ASE relies on sufficient SNPs differing between the parents [60]. Despite being inbred, neither the Kisumu nor the Nagongera parental strain was isogenic, consistent with previous observations of retained heterozygosity in inbred *A. gambiae* strains [62]. Nucleotide diversity and heterozygosity were lowest in the Kisumu colony and higher in the Nagongera colony although slightly reduced compared with wild mosquitoes caught in the same geographic area (electronic supplementary material, table S4). The number of SNPs differing between maternal and paternal genomes varied (table 2) and since the available SNPs considering each cross separately was far in excess of the consensus differences between the colonieswe used the SNP sets from each cross for further analysis. SNP level ASE was inferred for each cross (figure 3; electronic supplementary material, figure S2). For the four crosses where the parent was known, per gene ASE was calculated as reads mapping to the maternal genome/total reads mapped at that gene. In addition, the MAF at each gene was calculated for all crosses. Read count data were fitted to binomial and beta-binomial models. ANOVA indicated that the beta-binomial model was a better fit to the data (*p* < 2.2e−16). Per gene ASE statistics for all crosses are shown in table 3. Due to variations in parental genome and sequencing depth the power to detect ASE varies between crosses, so it is not possible to infer whether the proportion of genes with ASE is different between the crosses. Crosses B5, K2 and K4 showed similar numbers of SNPs (figure 3; electronic supplementary material, figure S2) and genes with maternal or paternal ASE but cross K6 showed extreme paternal ASE. This could either be due to K6 showing a very different pattern of ASE to the other crosses, or a sampling error occurred so that the RNA sequenced samples were not from the same cross as the parents and siblings. We therefore checked the effect of using SNPs from the wrong parents to infer ASE. Using SNPs from non-matching parents resulted in inflated ASE estimates (electronic supplementary materials, table S4 and figure S3). Using SNPs from the parents of cross K4 to infer allelic counts for cross K6 shifted mean imbalance back to 0.5 (figure 3; electronic supplementary materials, figure S2 and table S5). Furthermore, when SNPs called from the RNAseq data were compared, the progeny of cross K4 and K6 were extremely genetically similar and clustered closely in PCA for all chromosomal arms (electronic supplementary material, figure S4), suggesting they may have hatched from two egg batches from the same cross. Gene level ASE for cross K6 was therefore inferred using SNPs from K4 parents.

**Table 3.** Number of genes showing ASE in each cross.

Cross	Genes showing ASE (FDR 5%)	Total genes with SNP(s) and sufficient counts to detect ASE
B1	327	6486
B3	324	6065
B5	823	7537
K2	255	5185
K4	979	8166
K6	757	7852
All (intersection)	13	2934

Numbers are exonic SNPs that are homozygous in the parents of each cross but differing between the parents.

**Figure 4.**
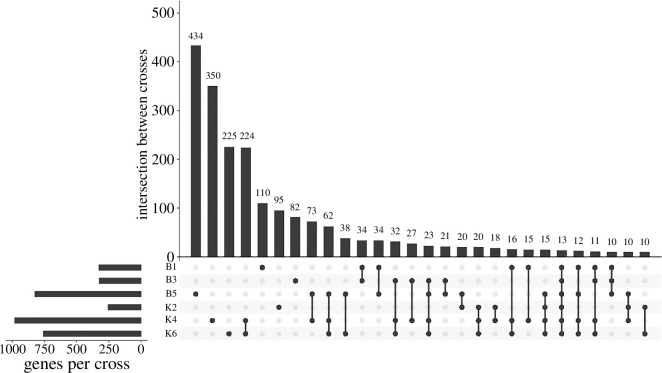
Intersection of genes showing ASE between crosses. UpSet plot with the number of genes showing ASE for each cross and the intersection of these genes between crosses. Only the first 28 sets of overlaps are shown.

Genes showing ASE showed some overlap between the different crosses (figure 4). At the most conservative estimate, 13 genes showed ASE in all crosses. This exceeds the overlap expected by random genes showing ASE, as in 100 000 simulations of randomly drawing the observed number of significant genes from the set of genes where SNPs were available to detect ASE in all crosses, the maximum overlap was 1. Most genes showing ASE were unique to individual crosses (figure 4), but many genes showed ASE in combinations of multiple crosses that may also be under consistent differential *cis*-regulation between Tororo and Nagongera colonies. Maternal or paternal ASE bias was inferred for crosses B5, K2, K4 and K6 (electronic supplementary material, tables S6 and S7). Table S6 shows the 115 genes with significant ASE in at least 4 out of the 6 crosses. Significant *p*_het_ was observed in at least one cross for 62 of these, suggesting possible isoform-specific ASE.

**Figure 5.**
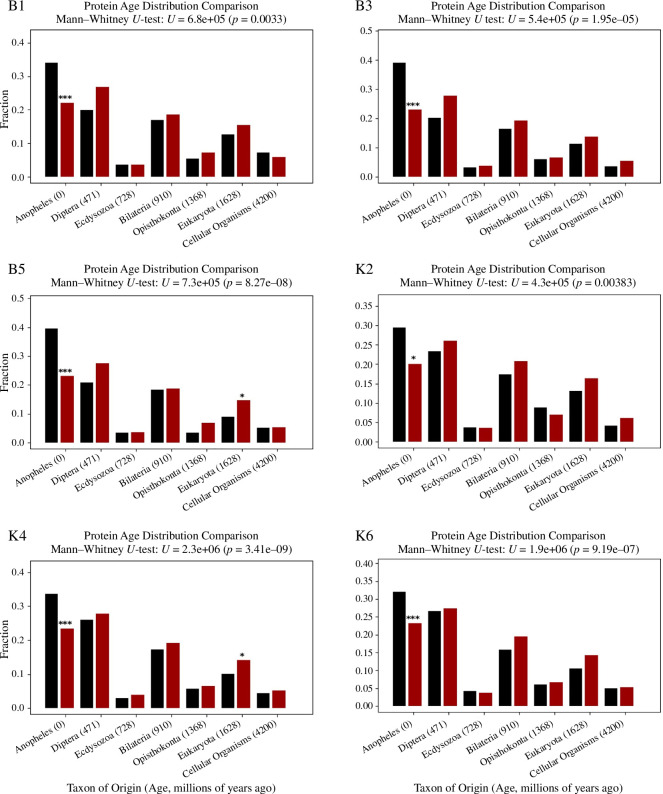
Ages of genes showing ASE or not showing ASE. Bar plots comparing the age of genes showing ASE (black bars) and without ASE (red bars) in the progeny of six crosses between Nagongera and Kisumu strains, using the Wagner parsimony method. The cross name is indicated at the top left of each plot, Fisher’s exact test *p*-values for the difference in fraction of genes in each age between ASE/non-ASE genes are displayed above the bars. *0.001 < *p* < 0.05; ***p ≤ 0.001.

Analysis of gene ages that showed or did not show ASE revealed an enrichment of younger, *Anopheles* specific genes showing significant ASE (figure 5; electronic supplementary material, table S8). The same trend was seen in all crosses.

Genes showing ASE showed an unequal distribution along chromosomal arms compared with genes not showing ASE (electronic supplementary materials, table S9 and figure S5). This trend was significant for all crosses except for K2. ANOVA indicated both chromosome and cross explained the variance in the proportion of ASE/total detectable genes per chromosome arm (*p* < 0.05).

### (d) Copy number variation

True ASE is caused by *cis*-regulation, but CNV of the expressed gene could lead to apparent ASE if there are different numbers of gene copies containing the SNPs used to count reads; e.g. if two copies of a duplicated gene with total of three copies bear one SNP and the third the other SNP, apparent ASE would be inferred without differential *cis*-regulation. We therefore checked for CNV in the parental and F1 sibling genomes at the genes that showed ASE in at least 4 out of 6 crosses, and in the genes which contained sufficient SNPs to infer ASE but expression appeared to be in balance. The number of individuals with a possible CNV in each gene varied between the genes (table S6) . A total of 60/114 (53%) autosomal genes showing ASE contained possible CNV. For genes that did not show ASE, 481/1333 (36%) had possible CNV. A two-sided Fisher’s exact test rejected the hypothesis that the odds ratio is 1 (odds ratio 2.0, *p* = 0.0006) suggesting that for some genes CNV contributed to the observed ASE. For example*, ace1* (AGAP001356) is the target site of bendiocarb, and a CNV in this gene has previously been implicated in resistance to bendiocarb [63]. AC0398-C, the Nagongera father of crosses K4 and K6, had three copies of *ace1*, as did some of the F1 progeny of these crosses. However, *ace1* ASE also occurred independently of the CNV as it was also observed in the progeny of cross K2 for which neither parent had a CNV in *ace1*.

### (e) Overlaps between ASE genes and other datasets

The list of 115 genes showing ASE in at least four crosses was compared with published gene expression data. All other available *Anopheles* expression data is based on between sample comparison and represents a mixture of *cis-* and *trans*-regulation, whereas ASE data is specific to *cis*-regulation and excludes any *trans*-regulation. Therefore even for extremely similar ASE and between sample comparison datasets only *cis*-regulated genes are expected to overlap.

Genes showing ASE in at least four crosses were compared with genes with consistently high median fold change in a meta-analysis of 35 experiments comparing RNAseq data between *A. gambiae* s.l. and *Anopheles funestus* strains [64], and with genes showing significant fold change with consistent directionality in microarrays comparing susceptible and resistant populations of *A. coluzzii* [65]. For the RNAseq metadata set, the genes with the top 5% of median fold changes between susceptible and resistant populations (429/8599 total genes in the dataset), six genes were also present in the ASE gene set. These were AGAP001251 (*Eupolytin*), AGAP008218 (*Cyp6Z2*), AGAP012296 (*Cyp9J5*), AGAP004534 (*cathepsin B precursor*), AGAP003583 (*L-iditol 2-dehydrogenase*) and AGAP008331 (*WD repeat-containing protein 59*). There was no overlap between ASE and microarray data.

Gene set enrichment analysis did not reveal any consistent patterns across crosses for the enrichment of genes showing significant ASE in any GO term or Kegg pathway. Trypsin and Ubiquitin pfam domains were significantly enriched in four of the six crosses.

Of the gene families previously implicated in metabolic resistance, we observed four P450s with ASE in four of the six crosses: *Cyp12F3* (AGAP008019), *Cyp12F2* (AGAP008020), *Cyp6Z2* (AGAP008212) and *Cyp9J5* (AGAP012296). Cy6Z2 and Cyp9J5 are overexpressed in strains from Burkina Faso which exhibit varying degrees of resistance to organochlorine, carbamate and pyrethroid insecticides [45]. Alpha-crystallins are chaperone proteins which bind denaturing proteins preventing their aggregation [66] previously implicated in deltamethrin resistance and response to insecticide exposure [65]. Alpha-crystallins AGAP005548 and AGAP007159 showed ASE in four of the six crosses.

The D7 protein family has previously been implicated in bendiocarb resistance in Uganda [25]. AGAP008282 (*D7r2*) showed significant ASE in two crosses (detectable in 4) and *D7r4* in none (detectable in 3). Other *D7r* genes, *D7r1* (AGAP008284), *D7r5* (AGAP008280) also showed significant ASE in two crosses, *D7L1*, *D7L2* and *D7r3* in one cross.

We finally asked whether genes showing ASE were more likely to be in a genomic region that has undergone a recent selective sweep (see electronic supplementary materials). Out of 115 genes showing ASE in at least four of the six crosses, 13 were in a swept region, whereas 103 of the 1333 genes showing no evidence of ASE in any of the six crosses were in swept regions. A two-sided Fisher’s exact test did not reject the hypothesis that the odds ratio is 1 (odds ratio 1.5, *p* = 0.2), suggesting that genes showing ASE are no more likely to be in a swept region than genes that do not.

### (f) CRM prediction

*Drosophila* CRMs for adult midgut, adult Malpighian tubules, larval midgut and legs were used as training data from SCRMshawHD to predict *Anopheles* CRMs operating in the same tissues, together with previously developed training sets for the adult peripheral nervous system, embryonic and larval excretory system and embryonic/larval Malpighian tubules (https://github.com/HalfonLab/dmel_training_sets). These tissues were selected based on previous studies examining the tissue-specific expression of genes involved in insecticide resistance with roles including detoxification and cuticular resistance [67–72]. Training CRM sets used for the first time in this study are shown in electronic supplementary material, table S10 (full sequences at https://github.com/azurillandfriend/traning_sets_IR.git). Oenocyte (FBbt:00004995), cuticle (FBbt:00004970) and adult epidermis (FBbt:00005401) CRMs could not be used as training data due to insufficient experimentally validated CRMs, highlighting the need for more research to identify CRMs in these tissues. The top scoring 250 predictions for each training set and method were combined, producing a total of 4122 unique CRM predictions [61]. Sixty-two predicted CRMs were flanked by a gene showing ASE in at least four of the six crosses (electronic supplementary material, table S11).

In total, CRMs were predicted for 33 of the 115 genes showing ASE in at least four of the six crosses. A total of 211 predicted CRMs were flanked by a gene showing consistently high median fold change between resistant and susceptible strains (electronic supplementary material, table S12). CRMs were predicted for a total of 141 of the 429 genes in this set. Predicted CRMs were also compared with a published dataset of Tn5 transposase sensitive sites in the adult midgut [59,73]. Fifty predicted CRMs overlapped with Tn5 transposase sensitive sites identified as *cis*-regulatory elements by Ruiz *et al.* [59], flanking a total of 48 genes. The majority of these CRMs were previously predicted by Kazemian *et al.* [74] despite the different training sets.

We hypothesized that CRMs responsible for ASE in F1 hybrids between Kisumu and Nagongera contain SNPs that differ between these colonies. We therefore located SNPs in the 62 predicted CRMs flanking genes showing ASE in at least 4 out of 6 crosses and which were homozygous in parents but different between the colonies. Such SNPs were found in 9 out of the 62 predicted CRMs (electronic supplementary material, table S13). It is possible that the CRMs contained other types of mutations such as insertions and deletions, and that only selecting SNPs which were present in all the parents filtered out some SNPs which could underlie ASE. Indeed there were a large number of other segregating SNPs in 38 of the CRMs [61]. The limited training CRMs for some insecticide resistant relevant tissues and lack of empirical data on *A. gambiae* CRMs operating outside of the midgut and salivary glands mean that there are likely many undiscovered CRMs regulating the genes showing ASE.

The CRMs identified in these predictions provide a starting point for future studies to examine genetic variation in *Anopheles* populations with different insecticide resistance phenotypes. Future research can now evaluate the contribution of genetic variants in CRMs to *cis-*regulation of gene expression using expression quantitative trait loci, targeted association studies and CRM reporter assays.

## 4. Conclusions

The study of ASE in *Anopheles gambiae* provides the first evidence that *cis*-regulation of gene expression occurs across the genome and differs between strains in this species.

Sample pooling, while a cost-effective solution for bulk RNAseq of small samples, limits the power of experiments targeting the study of ASE; in future, to maximize the power to detect ASE, individual samples should be used. RNAseq on whole mosquitoes may have masked tissue-specific ASE. The true number of genes showing ASE in individual tissues is likely higher than we observed on the whole mosquitoes analysed here, since pleiotropic effects may limit the potential for genes to upregulated or downregulated in all tissues simultaneously but permit the evolution of tissue-specific regulation [75]. Future studies should target specific tissues of interest. Despite these limitations, we were able to detect genes showing ASE in pooled RNA from whole mosquitoes in crosses between *Anopheles* strains with different carbamate resistance phenotypes, indicating different *cis*-regulation patterns between the strains. While we detected some genes previously implicated in insecticide resistance, there was no consistent enrichment of these or any particular GO term among genes showing ASE. This probably indicates that the strains have undergone extensive *cis*-regulatory divergence, affecting both genes involved in insecticide resistance but also genes involved in many other functions. Comparing ASE in progeny of crosses between multiple insecticide resistance and susceptible strains, together with examining gene expression in the parental strains, would enable a more comprehensive survey of the *cis*-regulation versus *trans*-regulation of insecticide resistance genes. The bias towards younger, *Anopheles* specific genes showing ASE in the F1 suggests there may be a higher degree of *cis*-regulatory divergence between the parental strains for younger genes.

It was possible to computationally predict some CRMs involved in tissue-specific expression, including potential CRM for genes showing ASE and genes previously implicated in insecticide resistance. This was hampered by the lack of good quality training data for the tissues thought to be most relevant to insecticide resistance in adult mosquitoes, highlighting the need for future experimental CRM discovery.

## Data Availability

RNAseq data for all crosses and read counts per SNP are available at Gene Expression Omnibus accession GSE241768 (https://www.ncbi.nlm.nih.gov/geo/query/acc.cgi?acc=GSE241768). Potential fathers sequencing data are available at European nucleotide archive (accession numbers in electronic supplementary material, table S14). Sequencing data from siblings from crosses B1 and B3 are at European nucleotide archive (accession numbers in electronic supplementary material, table S15). All other nucleotide sequences used are already publicly available in the Phase 3 release of the Anopheles gambiae 1000 genomes project, at accession PRJEB42254 (https://www.ebi.ac.uk/ena/browser/view/PRJEB42254). Individual accessions for the parents and F1 male sibs in crosses B5, K2, K4 and K6 are in electronic supplementary material, table S16. All the predicted CRMs, segregating sites in predicted CRMs flanking genes showing ASE and analysed ASE results for all crosses and genes are available at Dryad [61]. Supplementary material is available online [76].
